# A Comparison in Mechanical Properties of Cermets of Calcium Silicate with Ti-55Ni and Ti-6Al-4V Alloys for Hard Tissues Replacement

**DOI:** 10.1155/2014/616804

**Published:** 2014-11-04

**Authors:** Azim Ataollahi Oshkour, Sumit Pramanik, Seyed Farid Seyed Shirazi, Mehdi Mehrali, Yat-Huang Yau, Noor Azuan Abu Osman

**Affiliations:** ^1^Department of Mechanical Engineering, Faculty of Engineering, University of Malaya, 50603 Kuala Lumpur, Malaysia; ^2^Department of Biomedical Engineering, Faculty of Engineering, University of Malaya, 50603 Kuala Lumpur, Malaysia

## Abstract

This study investigated the impact of calcium silicate (CS) content on composition, compressive mechanical properties, and hardness of CS cermets with Ti-55Ni and Ti-6Al-4V alloys sintered at 1200°C. The powder metallurgy route was exploited to prepare the cermets. New phases of materials of Ni_16_Ti_6_Si_7_, CaTiO_3_, and Ni_31_Si_12_ appeared in cermet of Ti-55Ni with CS and in cermet of Ti-6Al-4V with CS, the new phases Ti_5_Si_3_, Ti_2_O, and CaTiO_3_, which were emerged during sintering at different CS content (wt%). The minimum shrinkage and density were observed in both groups of cermets for the 50 and 100 wt% CS content, respectively. The cermets with 40 wt% of CS had minimum compressive Young's modulus. The minimum of compressive strength and strain percentage at maximum load were revealed in cermets with 50 and 40 wt% of CS with Ti-55Ni and Ti-6Al-4V cermets, respectively. The cermets with 80 and 90 wt% of CS showed more plasticity than the pure CS. It concluded that the composition and mechanical properties of sintered cermets of Ti-55Ni and Ti-6Al-4V with CS significantly depend on the CS content in raw cermet materials. Thus, the different mechanical properties of the cermets can be used as potential materials for different hard tissues replacements.

## 1. Introduction

Calcium silicate (CS, CaSiO_3_) and titanium (Ti) and Ti alloys have widely been used in implants especially for bone hard tissue due to their unique bioactivity and biocompatibility properties [1]. CS is a highly bioactive ceramic, while it is a brittle material with low fracture toughness [2–4]. Therefore, many studies tried to increase the load bearing capacity and toughness of CS by reinforcing it with other materials such as alumina [5], carbon nanotube [6], graphene oxide [7], reduced graphene oxide [8], and polymers [9]. On the other hand, Ti and Ti alloys are biocompatible and have excellent corrosion resistance [1]. However, they are bioinert materials without having bioactivity to create a strong bonding with host bone as implant [10]. As a result, the Ti alloys implants encapsulated by fibrous tissue cause clinical failure due to isolation from the surrounding tissue after implantation into a living body [10, 11]. In this context, monolithic ceramics, particularly CS [2, 5] and synthetic or natural hydroxyapatite (HA) [12–15], are known as excellent biocompatible materials. Therefore, the metallic implants have been coated with some bioactive materials such as HA and CS to ensure a good bonding between implant and host bone [16–20]. However, coating could be flaked off from metals substrate because of weak bond between ceramic and metallic phases [21]. In addition, our several theoretical studies have shown that stress intensity factors at the external surface of the cement layer of the cement coated metallic implants are higher which may be the primary cause of easy flaking off of the coatings from the metallic surfaces [22, 23]. Composites of many ceramics, including HA [13, 24], CS [14, 25], and alumina (Al_2_O_3_) [5, 26], have been used in different biomedical applications successfully. Therefore, composites or cermets of Ti alloys with CS or HA ceramics could be employed to resolve the problems related to the coating and brittleness of pure ceramics.

The composite material made of the ceramics and metals is called cermet. Based on the matrix and the reinforcing materials, composite materials are categorized into the ceramic matrix-metal composites (CMMCs) and metal matrix-ceramic composites (MMCCs). A dramatic change in mechanical properties in cermets is a result of the ceramic or metal particles inclusion into the metal or ceramic matrix [27, 28]. Thus, due to the overlapping strength and weakness of the ceramics and metals, the CMMCs and MMCCs possess superior stiffness, fracture, fatigue, and tribological and thermal properties in comparison with the monolithic ceramic and metals counterparts  [29].   According to the suitable properties of conventional and monolithic materials, the monolithic ceramics and metals are rapidly changed with these composites in various engineering applications from biomedical [24, 26] to the aerospace or automobile fields [30].

Despite many works on Ti, Ti alloys, and CS in different applications according to their individual important properties, to the best of our knowledge there has been no work on composites of CS with Ti-55Ni (TN) or Ti-6Al-4V (TAV) reported. Therefore, in the present study, first time we investigate the effect of CS content on the mechanical properties and microstructural fracture surfaces in the TN/CS and TAV/CS cermets for hard tissues replacement in biomedical applications.

## 2. Materials and Methods

**Figure 1:**
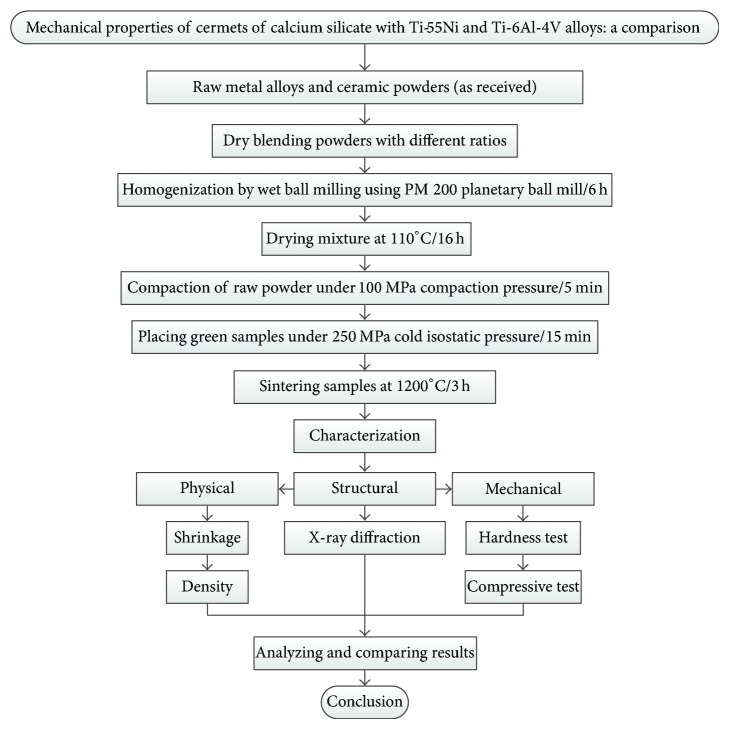
A schematic flowchart of all methods employed in the present work.

The procedure employed in the present work to study the cermets of calcium silicate with Ti-55Ni and Ti-6Al-4V is illustrated as a schematic flowchart in Figure 1.

### 2.1. Sample Preparation

**Figure 2:**
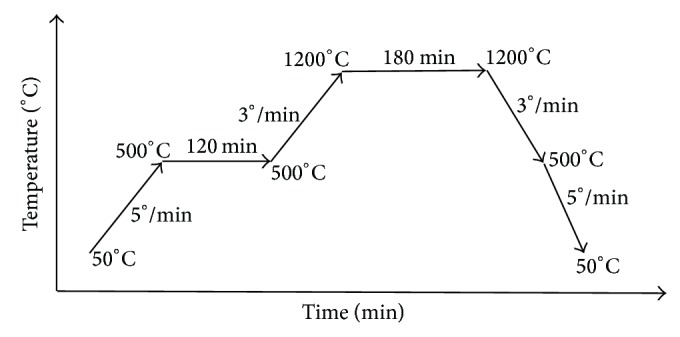
A schematic of sintering steps (the temperature and time scale factors are arbitrary).

Powder metallurgy technique was employed to fabricate the different novel cermets of TN and TAV with variation of CS contents in two different cermets in the form of ceramic/metal matrix composites and vice versa. All the chemicals were purchased from Sigma-Aldrich and used without any further purification. Briefly first, the raw powders were mixed in different weight ratios as presented in Table 1. Then wet ball milling was carried out in a planetary ball mill (PM 200, Retsch) to blend the raw powders at a ball to powder weight ratio of 5 : 1 in 75 mL ethanol medium at a speed of 300 rpm for 6 h to get a homogeneous mixture. The mixture was dried overnight in an oven at temperature of 110°C for 16 h. Following that, the dried milled powders were compacted under 100 MPa by using manual hydraulic press (GS15011, Graseby Specac) to form green samples of fixed diameter of 6.35 mm and average compact height of 13 mm. Afterwards, the green compacted samples were placed in cold isostatic pressure (CIP) machine (Reiken Seiki Japan) at room temperature under pressure of 250 MPa to achieve further densification and a uniform compacting pressure distribution. Then pressureless sintering was performed on samples at 1200°C for 3 h in inert argon gas environment using a vacuum atmosphere furnace (XY1600, Nanyang Xinyu Furnaces) to prevent oxidation of metal phases. Sintering cycle exploited in this study is presented in Figure 2. The temperature was increased gradually at rate of 5°C/min from 50°C to 500°C. Then the temperature was kept at a constant holding temperature of 500°C for 2 h to improve the sintering ability in addition to removing the moisture and some impurities of the samples. After that the temperature was again increased at rate of 3°C/min to 1200°C and was soaked for 3 h. The ramp rate was decreased in the second step to prevent the crack formation in the samples owing to the thermal shock as well as difference in the thermal expansion coefficients of the phases present in the cermets. The 3h soaking time at 1200°C was used to complete the sintering process to the whole sample. Afterwards, the temperature was cooled to room temperature with two steps of 3°C/min and 5°C/min ramp rate, respectively, to avoid the crack formation as well as thermal stress in the samples.

### 2.2. Characterization

X-ray diffraction (XRD) was conducted on sintered samples by using X-ray diffractometer (Empyrean, PANalytical) to determine and analyze the phase constitution. The density (*ρ*, g/cc) was measured by employing Archimedes' principle explored elsewhere [25] according to (1) and volume shrinkage (*ϵ*%) of sintered samples was computed following (2). The materials were assumed to be perfectly solid and completely insoluble in water at the measured condition. At least five identical specimens were carried out at 25°C, where water density was considered as 0.99704 g/mL, to compute the average as well as standard deviation (SD) for each sintered sample:

(1) $$\begin{array}{c} \text{Density}\left(\rho \right)=\frac{{\text{Weight}}_{\text{in}\;\text{air}}}{{{\text{Weight}}_{\text{in}\;\text{air}}-\text{Weight}}_{\text{in}\;\text{water}}}\times \text{Water}\;\;\text{density}, \end{array}$$

(2) Volume  Shrinkageϵ=Initial  Volume  −Final  Volume  Initial  Volume ×100%.

**Figure 3:**
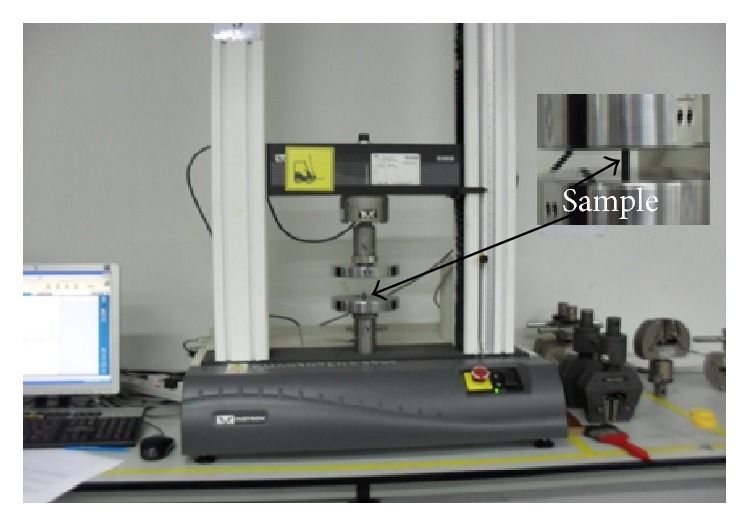
Instron universal testing machine used for compression test.

The compressive test conducted on samples using universal testing machine (4469, Instron) at a constant cross-head speed of 1 mm/min to determine compressive elastic modulus, compressive strength, and strain percentage at maximum load is shown in Figure 3. At least three identical samples were performed to take the mean and SD of mechanical properties.

**Figure 4:**
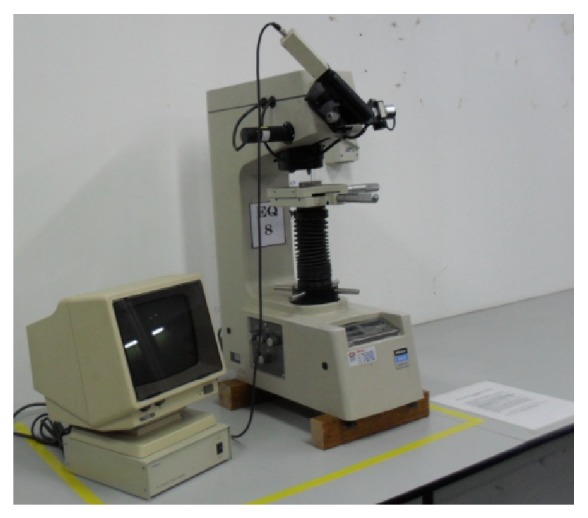
Microhardness tester machine.

The Vickers hardness test was conducted on the polished surfaces of sintered samples using micro-Vickers hardness tester (AVK-C200, Mitutoyo) as shown in Figure 4. The pyramid shaped diamond indentor was used at constant load of 5 N for 10 seconds to take at least 5 indentations from each sample.

**Figure 5:**
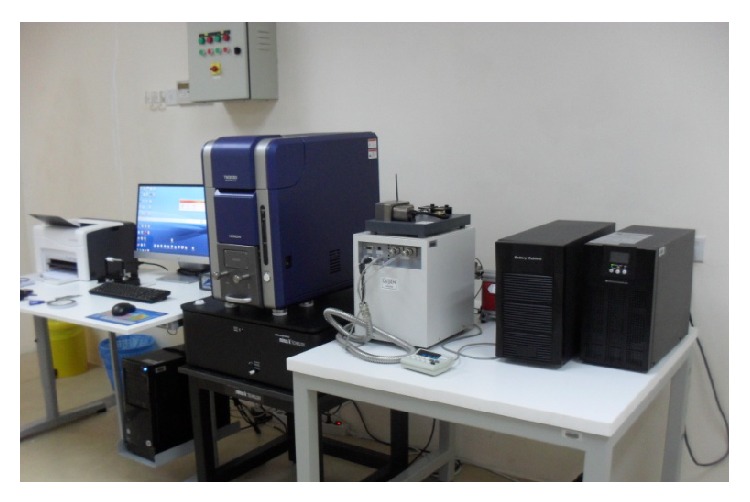
Hitachi scanning electron microscope.

Finally, the microstructure of sintered samples was examined by scanning electron microscope (TM3030, Hitachi) as shown in Figure 5.

## 3. Results and Discussion

### 3.1. Structure Characterization

**Figure 6:**
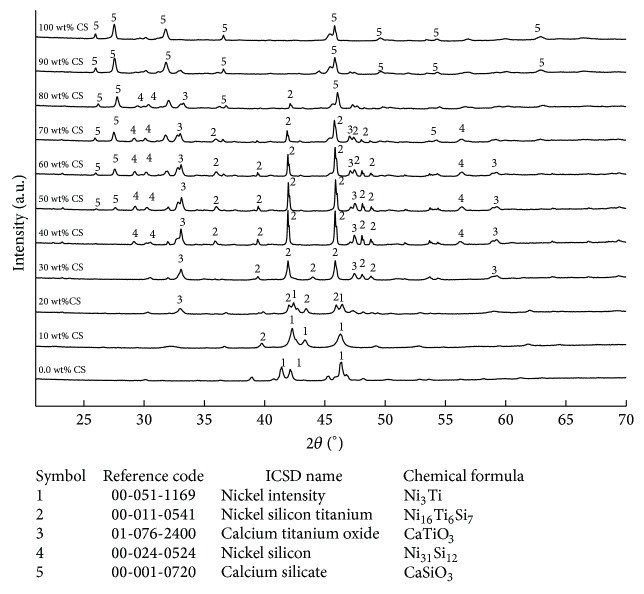
XRD patterns of Ti-55Ni/CaSiO_3_ sintered cermets with different CS contents. New phases are revealed from 10 to 80 wt% CS contents in TN/CS cermets.

**Figure 7:**
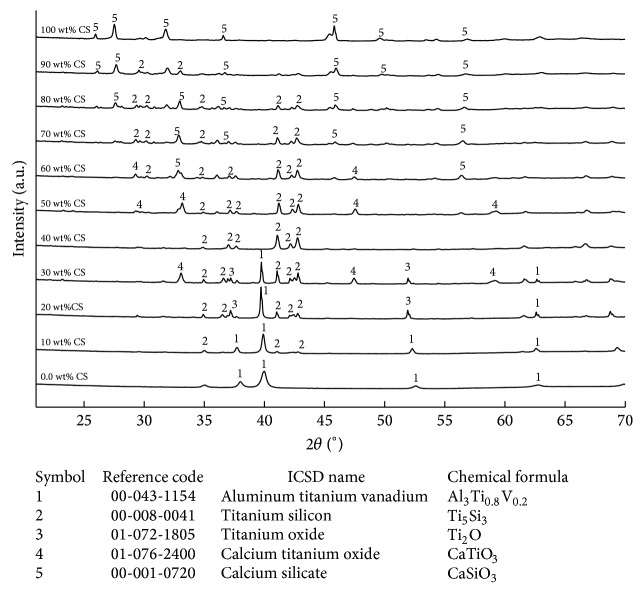
XRD patterns of Ti-6Al-4V/CaSiO_3_ sintered cermets with different CS contents. New phases are revealed from 10 to 90 wt% CS contents in TAV/CS cermets.

Figures 6 and 7 depict the XRD patterns of different cermets of the TN/CS and TAV/CS after sintering at 1200°C, respectively. In sintering process, three new phases of nickel silicon titanium (Ni_16_Ti_6_Si_7_), calcium titanium oxide or calcium titanate (CaTiO_3_), and nickel silicon (Ni_31_Si_12_) appeared in XRD patterns of TN/CS cermets (see Figure 6). These phases were the results of reaction between compounds of TN and CS. The volume fraction of new phases in sintered cermets was a function of CS wt%. With increasing of CS content in TN/CS cermets, the crystalline peaks of  Ni_16_Ti_6_Si_7 _clearly appeared in the cermets from 10 to 80 wt% CS, while the CaTiO_3 _phase appeared from 20 to 80 wt% and the Ni_31_Si_12 _phase was found mainly from 40 to 80 wt% CS in XRD patterns of Figure 6.

On the other hand, three new phases of titanium silicon (Ti_5_Si_3_), titanium oxide (Ti_2_O), and CaTiO_3_ were also developed in another sintered cermet (TAV/CS) comprised TAV and CS as shown in Figure 7.   It has also been found that few of the XRD peaks in pure TAV (i.e., 0.0 wt% CS) also match the titanium oxide (Ti_3_O, ICSD reference code: 01-072-1806). This trace amount of Ti_3_O is produced due to the oxidation of a small part of the surface. The Ti_5_Si_3 _phase was found in all cermet groups of TAV with 10 to 90% wt of CS content. CaTiO_3_ was revealed in sintered cermets with more than 30 wt% CS except in group of 40 wt% CS which showed only phase of Ti_5_Si_3_. On the other hand, Ti_2_O only was found in groups of TAV with 10 wt% and 20 wt% CS. The different extra phases developed in both the cermets are mainly responsible for the mechanical properties, such as compressive strength and microhardness, and density or shrinkage of the sintered cermets.

### 3.2. Physical Characterization

**Figure 8:**
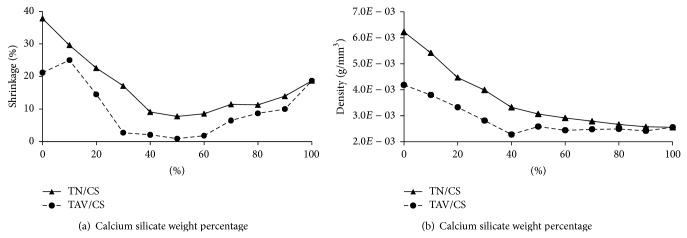
(a) Shrinkage and (b) density of sintered samples of the cermets Ti-55Ni/CaSiO_3 _(TN/CS) and Ti-6Al-4V/CaSiO_3_ (TAV/CS).

Figures 8(a) and 8(b) represent shrinkage and density of different cermets of TN and TAV with CS after sintering process. The variation of shrinkage as a function of CS wt% shows a concave plot with the maximum value of shrinkage in the pure metallic and ceramic phases and a minimum value at around 50 wt% of  CS in both groups of cermets (see Figure 8(a)). The percentage of shrinkage is nearly constant in 40 to 60 wt% CS contents for both cermets, while a dramatic increase in shrinkage has been found below 40 wt% CS and a gradual increment is noticed for the cermets above 60 wt% CS content. On the other hand, increase of CS content in cermets leads to gradual reduction in density of sintered cermets samples.

Therefore, pure metallic phase showed the maximum density and pure ceramic phase showed minimum density (Figure 8(b)). In contrast, the reduction in density with increase of CS content was consistent with a study of Chenglin et al. [21] for Ti-HA functionally graded materials. In addition, the density of pure CS used in the current study is in the range of density reported by Liu et al. [31].

### 3.3. Mechanical Characterization

**Figure 9:**
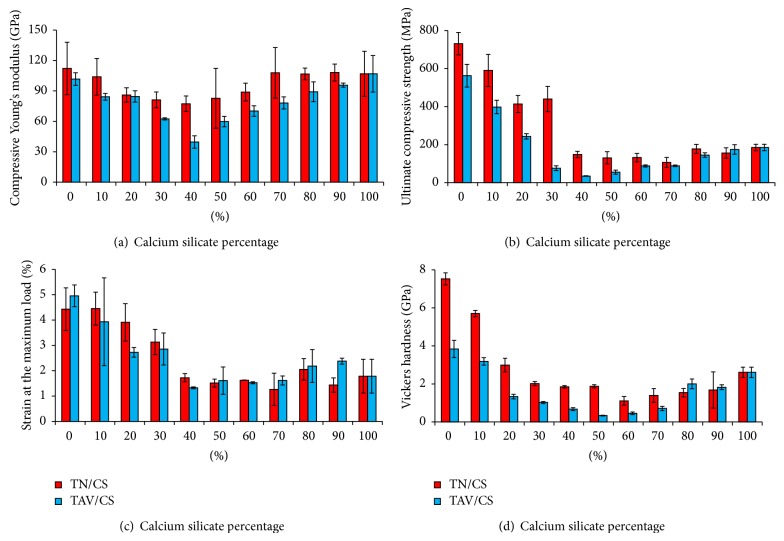
Mechanical properties of  Ti-55Ni/CaSiO_3 _(TN/CS) and Ti-6Al-4V/CaSiO_3 _(TAV/CS); (a) compressive Young's modulus; (b) ultimate compressive strength; (c) strain at maximum load; (d) Vickers hardness.

In order to investigate the relationship between CS content and the mechanical properties of sintered specimens, compressive properties were measured and the results were presented as a function of CS content (wt%) in Figure 9.  Figure 9(a) depicts variation of Young's modulus in different cermet groups of TN/CS and TAV/CS. The elastic modulus of cermet materials in both groups gradually decreased with CS content up to 40 wt% and then it followed an increasing trend up to pure CS (100 wt% CS). The TN/CS cermets group showed higher modulus elasticity than the cermets of TAV/CS in any CS content. An insignificant effect was noticed in the change in Young's modulus owing to the increase of CS content more than 70 wt% in TN/CS cermets. Based on the XRD patterns (Figure 6), the reason for limited change in modulus of elasticity in TN/CS cermet group with more than 70 wt% CS could be explained by dominance of unreacted CS on other phases.  Figure 9(b) compares the ultimate compressive strength (UCS) of different cermet groups of TN/CS and TAV/CS. The UCS demonstrated a similar trend as modulus of elasticity with respect to CS content. The metallic phases carried out more compressive load than the other cermets. In addition, the TN/CS cermet groups performed better under compressive load than the TAV/CS cermet group. The strain percentage at maximum load is shown in Figure 9(c). The trend in strain percentage as function of CS content (wt%) increase was similar to aforementioned properties. Strain of the cermets gradually declined with increase in CS content up to 30 wt%. Afterwards, a sharp reduction was found in strain at 40 wt% CS and then it increased with a low slope up to 70 wt% CS. At the 80 wt% and 90 wt% CS, more strain percentage at maximum load larger than the pure CS (100 wt% CS) was revealed. It indicates more plasticity in the groups of 80 wt% and 90 wt% CS for both cermets in comparison with their monolithic ceramic phase. The cermets with 60 wt% metallic phase and 40 wt% CS showed minimum ultimate compressive strength (UCS), while both the cermets with 70 wt% metallic phase and 30 wt% CS showed high UCS, excellent modulus of elasticity and maximum strain at UCS. In this study, it has been found that the main responsible phases for increasing the mechanical properties in case of  TN/CS and TAV/CS cermets are Ni_16_Ti_6_Si_7_ and Ti_5_Si_3_, respectively. In a special case of TAV/CS cermet at 30 wt% CS, the UCS is significantly lower compared to 30 wt% TN/CS which is a result of the development of more amount of a new CaTiO_3 _phase. The amount of increase in mechanical properties of above 50 wt% CS cermets is lower than that of the cermets below 50 wt% CS. The micro-Vickers hardness test result portrayed in Figure 9(d) also showed similar pattern as found in compressive properties. Results of Vickers hardness test showed that the pure metallic phases of each group of cermets had maximum hardness and gradually it decreased with CS content till 60 wt% in TN/CS and 50 wt% in TAV/CS and following that hardness of both cermets was found to be increased till 100 wt% CS. The hardness behaviour in study is in agreement with hardness trend in Ti-HA functionally graded material in works conducted by Watari et al. [32] and Chenglin et al. [21]. The Vickers hardness of 100 wt% CS is comparable with reported hardness for pure CS by Liu et al. [31]. Since the two new phases such as Ni_16_Ti_6_Si_7_ and Ti_5_Si_3_ were significantly found on the surface for both the TN/CS and TAV/CS cermets between 40 and 60 wt% CS, respectively, in XRD patterns, the overall mechanical properties were found to be significantly detracted to those cermets.

### 3.4. Scanning Electron Microscope

**Figure 10:**
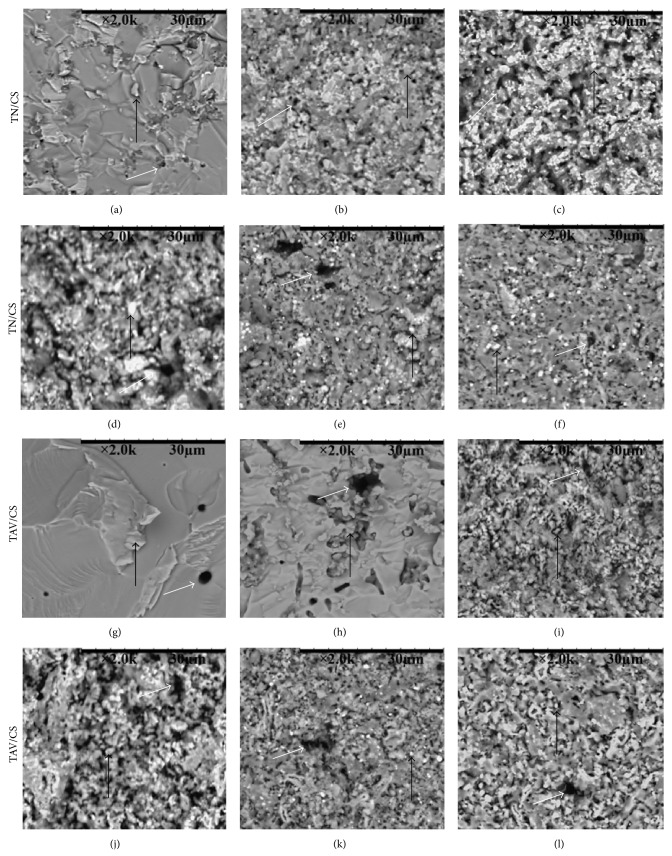
Scanning electron microscope (SEM) on fractured surface of the cermets TN/CS (top) and TAV/CS (down) with CS content (wt %) of (a and g) 0, (b and h) 10, (c and i) 30, (d and j) 50, (e and k) 80, and (f and l) 90. All the bars are 30 *µ*m; all black coloured arrows indicate the second phase particles and all white coloured arrows imply the pores at the fracture surface.

The SEM was conducted on samples after compressive test and some important specimens are selected to explore the fracture surfaces in Figure 10. The SEM images displayed the uniform distribution of CS in cermets. The sintered samples of  TN/CS showed less porosity than the TAV samples. The second phase particles and the pores at the fracture surface of the materials are indicated by black and white coloured arrows, respectively, on the SEM images in Figure 10. The fractured surfaces became rougher with increase in CS content up to 50 wt% CS in both groups of cermets and then the roughness of fractured surfaces gradually declined with more increase in CS content. The smooth surface for fractured surfaces reveals brittle facture, more bonds near to the pure phases of cermets in comparison with the cermets close to the 50 wt% CS.

## 4. Conclusion

During the sintering process at 1200°C based on the weight ratio of raw powders, different new phases were produced in final product during sintering process. The maximum shrinkage was revealed in pure metallic phases (TN and TAV) and ceramic phase (CS), while the minimum shrinkage was revealed at 50 wt% CS in different cermets of TN/CS and TAV/CS. On the other hand, density of cermets declined by CS weight percent increase and 100 wt% CS showed the minimum density. The new phases of  Ni_16_Ti_6_Si_7_, CaTiO_3_, and Ni_31_Si_12_  appeared in TN/CS cermets and Ti_5_Si_3_, Ti_2_O, and CaTiO_3_ in TAV/CS cermets are mainly responsible for their mechanical properties. The compressive mechanical properties and hardness decreased with CS content up to 50 wt% and then followed an increasing up to 90 wt% CS. The amount of increasing in overall mechanical properties of above 50 wt% CS cermets was lower than that of the cermets below 50 wt% CS. The TN/CS illustrated better compressive properties than TAV/CS. In addition, the strain percentage at the maximum load for cermets with 80 wt% and 90 wt% CS was more than pure ceramic phase of CS. Since the mechanical properties of the above cermets are substantially higher than that of the natural bones [25, 33], the different mechanical properties of the above cermets, therefore, can be used as per desired strength of the different hard tissues in biomedical applications [8, 33].

## Figures and Tables

**Table 1:** Weight percentage of different phases present in the cermets Ti-55Ni/CaSiO_3_ (TN/CS) and Ti-6Al-4V/CaSiO_3_ cermets (TAV/CS).

Ti-55Ni/CaSiO_3_ (TN/CS) cermets		Ti-6Al-4V/CaSiO_3_(TAV/CS) cermets	
TN Wt%	CS Wt%	TAV Wt%	CS Wt%
100	0	100	0
90	10	90	10
80	20	80	20
70	30	70	30
60	40	60	40
50	50	50	50
40	60	40	60
30	70	30	70
20	80	20	80
10	90	10	90
0	100	0	100
